# Recurrent association between *Trichodesmium* colonies and calcifying amoebae

**DOI:** 10.1093/ismeco/ycae137

**Published:** 2024-11-04

**Authors:** Futing Zhang, Siyuan Wang, Anna-Neva Visser, Coco Koedooder, Meri Eichner, O Roger Anderson, Sonya T Dyhrman, Yeala Shaked

**Affiliations:** The Fredy and Nadine Herrmann Institute of Earth Sciences, Hebrew University of Jerusalem, The Edmond J. Safra Campus, Jerusalem 9190401, Israel; The Interuniversity Institute for Marine Sciences in Eilat, Coral Beach P.O.B 469, Eilat 8810302, Israel; The Fredy and Nadine Herrmann Institute of Earth Sciences, Hebrew University of Jerusalem, The Edmond J. Safra Campus, Jerusalem 9190401, Israel; The Interuniversity Institute for Marine Sciences in Eilat, Coral Beach P.O.B 469, Eilat 8810302, Israel; The Fredy and Nadine Herrmann Institute of Earth Sciences, Hebrew University of Jerusalem, The Edmond J. Safra Campus, Jerusalem 9190401, Israel; The Interuniversity Institute for Marine Sciences in Eilat, Coral Beach P.O.B 469, Eilat 8810302, Israel; Friedrich-Alexander-University Erlangen-Nuernberg (FAU), Department Geographie und Geowissenschaften, Erlangen 91054, GeoZentrum Nordbayern, Schlossgarten 5, Germany; The Fredy and Nadine Herrmann Institute of Earth Sciences, Hebrew University of Jerusalem, The Edmond J. Safra Campus, Jerusalem 9190401, Israel; The Interuniversity Institute for Marine Sciences in Eilat, Coral Beach P.O.B 469, Eilat 8810302, Israel; Israel Limnology and Oceanography Research, Tel-Shikmona P.O.B 8030, Haifa 3108000, Israel; CentreAlgatech, Institute of Microbiology of the Czech Academy of Sciences, Novohradská 237, Třeboň 37901, Czech Republic; Biology and Paleo Environment, Lamont-Doherty Earth Observatory of Columbia University, 61 Route 9W, Palisades, NY 10964, United States; Biology and Paleo Environment, Lamont-Doherty Earth Observatory of Columbia University, 61 Route 9W, Palisades, NY 10964, United States; Department of Earth and Environmental Sciences, Columbia University, 1200 Amsterdam Avenue, New York, NY 10027, United States; The Fredy and Nadine Herrmann Institute of Earth Sciences, Hebrew University of Jerusalem, The Edmond J. Safra Campus, Jerusalem 9190401, Israel; The Interuniversity Institute for Marine Sciences in Eilat, Coral Beach P.O.B 469, Eilat 8810302, Israel

**Keywords:** *Trichodesmium*, colony, amoebae, association, *Trichosphaerium*, interaction, buoyancy

## Abstract

Colonies of the N_2_-fixing cyanobacterium *Trichodesmium* spp. constitute a consortium with multiple microorganisms that collectively exert ecosystem-level influence on marine carbon and nitrogen cycling, shunting newly fixed nitrogen to low nitrogen systems, and exporting both carbon and nitrogen to the deep sea. Here we identify a seasonally recurrent association between puff colonies and amoebae through a two-year survey involving over 10 000 *Trichodesmium* colonies in the Red Sea. This association was most commonly found in near-shore populations during spring. Microscopic observations revealed consistent amoebae morphology throughout the study, and both morphological characteristics and 18S rRNA gene sequencing suggested that these amoebae are likely to belong to the species *Trichosphaerium micrum*, an amoeba that forms a CaCO_3_ shell. Co-cultures of *Trichosphaerium micrum* and *Trichodesmium* grown in the laboratory suggest that the amoebae feed on heterotrophic bacteria and not *Trichodesmium*, which adds a consumer dynamic to the complex microbial interactions within these colonies. Sinking experiments with fresh colonies indicated that the presence of the CaCO_3_-shelled amoebae decreased colony buoyancy. As such, this novel association may accelerate *Trichodesmium* sinking rates and facilitate carbon and nitrogen export to the deep ocean. Amoebae have previously been identified in *Trichodesmium* colonies in the western North Atlantic (Bermuda and Barbados), suggesting that this type of association may be widespread. This association may add a new critical facet to the microbial interactions underpinning carbon and nitrogen fixation and fate in the present and future ocean.

## Introduction

Nitrogen limits phytoplankton growth and the biological fixation of carbon dioxide over large regions of the surface ocean, particularly the subtropical ocean gyres [1]. As such, nitrogen-fixing cyanobacteria play a key role in ocean ecosystem functioning by providing so-called “new” nitrogen to microbial communities. The cyanobacterium *Trichodesmium* spp. is such a keystone diazotroph, contributing up to half of the global oceanic dinitrogen (N_2_) fixation by some estimates [2, 3]. *Trichodesmium* occurs in tropical and subtropical regions and can form large surface blooms in both coastal and off-shore regions [4]. It occurs as filaments (trichomes) that often aggregate to form millimeter-sized colonies organized in fusiform (tuft) or spherical (puff) shapes [5]. *Trichodesmium* colonies, composed of hundreds of trichomes [6], have gas vesicles and can modulate their buoyancy to keep cells suspended in surface sun-lit waters [7]. Despite this ability to control buoyancy, recent work has identified *Trichodesmium* in sinking particulate matter [8, 9] that has intensified focus on the role this genus plays in the fixation and flux of marine nitrogen and carbon.

*Trichodesmium* colonies represent intricate microhabitats in pelagic ecosystems, and a vast range of bacteria and microeukaryotes differing in size and nutritional modes (i.e., heterotroph, phototroph, and mixotroph) have been observed associated with *Trichodesmium* colonies [10–12]. The *Trichodesmium* microbiome is distinct from sinking particles and free-living communities [10, 13], and the heterotrophic microbiome is tightly tuned to *Trichodesmium* physiological ecology [14–16], influencing patterns of resource cycling [12] and rates of nitrogen fixation [17]. These interactions suggest that *Trichodesmium* colonies could be considered a holobiont [18].

Over five decades, microscopic observations were the key tool to study colony associations with diverse micro-eukaryotic phytoplankton and zooplankton, fungi, and amoebae [10, 12]. More recently, advances in nucleic acid sequencing and bioinformatics have been applied for identifying the taxonomy of associated microorganisms (primarily prokaryotic bacteria) and their metabolic interactions with *Trichodesmium* [19–21]. The limited eukaryotic reference database and their relatively large and complex genomes [22] complicate the research on the taxonomy and metabolic interactions of colony-associated eukaryotes. While heterotrophic bacteria are now considered an integral part of the *Trichodesmium* colony consortium [23], microeukaryote associations with colonies are not well understood and have typically been hypothesized as transient observations and not necessarily reflective of a long-term association with ecological or biogeochemical significance [12, 13]. Although observations are relatively limited, *Trichodesmium* colonies have previously been reported to harbor amoebae [10, 12, 24].

Amoebae are microeukaryotes easily recognized by distinct amoeboid movement largely involving cytoplasmic extensions called pseudopodia [25]. Amoeboid organisms are not from a monophyletic taxonomic group, but mainly belong to the taxa Amoebozoa and Rhizaria and a few to the lineages of Metazoa and Fungi [26]. Amoebae typically feed when attached to suspended flocs or particles, preying primarily on bacteria but can also consume algae and microeukaryotes [27]. These particle- and colony-associated consumers are predicted to play a critical role in modulating microbiomes, and the recycling of resources like carbon and nitrogen when particles or colonies are exported to the deep sea [12]. Amoebae abundances in marine systems vary substantially with water depth, physico-chemical properties of the water mass, and the presence of suspended particles sufficiently large for the amoebae to attach upon and feed on bacteria [27]. Under favorable environmental conditions with plentiful particles, amoebae can be abundant, reaching up to a million cells per liter [27]. Amoebae are traditionally divided into naked forms (gymnamoebae) and testate forms (with a shell or so called test) [28]. The amoeba life cycle is highly complex and diverse, including dormant cysts, sexual stages and/or spore-bearing structures, which complicates studying their ecological dynamics in marine systems [29].

Despite the diversity of organisms that have been identified to be associated with *Trichodesmium* colonies, much recent research has focused on the *Trichodesmium* epibiotic prokaryotes, but not on its potential associations with microeukaryotes like amoebae [21, 30]. Given this knowledge gap, studies directed at *Trichodesmium*-microeukaryote associations are critical for expanding our understanding of microbial ecology and nutrient biogeochemistry of a keystone genus. In this study, we document a novel, recurrent association of a calcifying amoeba with *Trichodesmium* colonies in the field, and characterize its ecological dynamics through co-culture experiments.

## Materials and methods

### Observations of *Trichodesmium*-amoeba associations in the natural environment

*Trichodesmium* spp. colonies were sampled from the Gulf of Aqaba in the Red Sea (29.56°N, 34.95°E) throughout spring (April–July) and autumn (October–December) of 2021 and 2022. Open-water colonies were collected from 10–20 m depth using a 100-μm pore size plankton net towed by a motorboat. All tows were conducted at locations with a bottom depth of 300–400 m, 2–3 km from the shore. To enhance colony survival, net tows were kept short (7 min) and at a low speed (1–2 knots). Nearshore colonies were collected from 1–2 m depth using a 200-μm pore size plankton net placed statically at the pier of the Interuniversity Institute for Marine Sciences in Eilat (IUI) at a bottom depth of 3–4 m. Colonies were quickly handpicked using plastic droppers and suspended in Petri dishes containing fresh, 0.22-μm sterile-filtered seawater (FSW) from the point of collection. Following picking, colonies were examined using a stereoscope (Nikon SMZ745, Japan), separated into different morphotypes, counted, and washed by three successive transfers into new Petri dishes with FSW. The presence or absence of amoebae was documented using the stereoscope and a compound microscope (Nikon Eclipse Ci-E). Total associated amoeba numbers per colony were determined through microscope enumeration (see Supplementary 1.1 and Fig. S1). The density of the open-water *Trichodesmium* colonies was estimated according to the average volume of filtered seawater in the net tows as described in [31]. Sea surface temperature (SST) and chlorophyll *a* (Chl*a*) data in the Gulf of Aqaba were measured by the Israel National Monitoring Program at the Gulf of Eilat (NMP) (https://iui-eilat.huji.ac.il/Research/NMPMeteoData.aspx).

### Assessing possible impacts of associated amoebae on *Trichodesmium* colonies

#### Colony’s morphology and health

The physiological condition of natural colonies associated with amoebae was examined under the stereoscope during a 24-h incubation (Fig. S2). Colonies were assessed as either healthy, with dark-brown pigmentation and intact trichomes, or unhealthy, with light green pigmentation and broken or senescent trichomes [10]. Additionally, morphological changes were documented, which encompassed colony separation into free trichomes or splitting into smaller colonies.

#### Colony’s buoyancy

Sinking experiments were conducted in 18-cm tall chambers containing 100 mL FSW [32]. Fresh colonies collected on different days in 2022 were individually and gently placed at the water surface of the fully filled chamber and their vertical positions were recorded at three discrete time points (5, 10, and 15 min). Separately, the buoyancy data were related to the total amoebae weight of individual colonies, which was calculated by multiplying the counted amoeba numbers by their average cell density and cell volume (see the later section *Cell size, density, and nuclei number*) [33].

#### Colony’s bacterial assemblage

The bacterial diversity of *Trichodesmium* colonies was assessed through high-throughput sequencing of partial 16S rRNA gene (V4 region). Total genomic DNA was extracted from 30 colonies per sample using the QIAamp UCP DNA Micro Kit (Qiagen, Germany) (see Supplementary 1.2 for details). The sequenced data were analyzed with the QIIME 2 pipeline (v2022.11) [34]. Barcodes and primers were removed, and then the raw reads were merged, quality-filtered and de-noised using DADA2 [35]. The amplicon sequence variants (ASVs) were obtained and rarefied for downstream analysis. Representative sequences for ASVs were assigned to taxonomic categories using a trained Naive Bayes classifier based on the SILVA database release 138 [36]. Beta diversity was calculated using Unweighted UniFrac distance matrices, and the significance of bacterial community differences across groups was assessed through permutational multivariate analysis of variance (PERMANOVA) in QIIME 2 [37].

### Isolation and cultivation of amoebae

#### Amoebae isolation

Actively growing amoebae were successfully obtained from the *Trichodesmium* colonies and maintained in the laboratory by incubating individual natural amoebae-containing colonies for 1–3 months (Table S1). Such conditions resulted in a gradual decline and death of *Trichodesmium* [31] that was followed by state transformation and growth of the amoeba. These incubations consisted of 52 amoebae-containing and 30 amoebae-free colonies collected on different days in 2022, washed three times with FSW and individually placed in wells of sterile 48-well plates containing 1 mL FSW. Additional incubations of amoebae that were separated from the colonies were also attempted (see Supplementary 1.3 and Table S1).

#### Amoeba culturing

The wells yielding substantial amoeba growth were selected and the respective amoeba were cleaned by repeated resuspension in FSW, and then transferred to two different media. The first was bacteria-rich rice medium commonly used to grow heterotrophic protists (https://www.protocols.io/view/rice-medium-j8nlk5edwl5r/v1). The second was *Trichodesmium erythraeum* (strain IMS101) cultures that were grown with YBCII medium [38]. The culture was not axenic and as *Trichodesmium* ages, bacteria numbers increase substantially [39]. The amoeba cultures were transferred to new IMS101 cultures or rice medium every month for over a year.

Sterile techniques were applied for the experimental manipulations of both amoebae isolation and culturing. Individual colony incubations and amoeba cultures were placed in an algal growth chamber (WTC Binder, Germany) at 25°C and ~80 μmol photons m^−2^ s^−1^ (14 h:10 h light–dark cycle).

### Amoebae characterization and molecular identification

#### Amoeba 18S rRNA gene amplification and identification

Near-complete amoeba 18S rRNA gene sequences were amplified using universal eukaryote primers RibA and RibB [40], with template DNA from two independent amoeba cultures (Amoe_Colony1 and Amoe_Colony2, Table S2). The detailed procedures are provided in the Supplementary 1.4. The obtained sequences were edited and assembled using BioEdit, and aligned with ClustalW. To identify the closest amoeba, BLASTn searches were conducted in the NCBI database.

#### Cell size, density and nuclei number

Cell diameters were determined based on images of fresh amoebae-containing colonies observed using a 40x objective of the microscope and were converted to volumes using the equation for a sphere. Average cell weight was estimated by weighing a high number of Amoe_Colony1 culture (Table S2) using a microbalance (see Supplementary 1.5) [33]. Images of cell nuclei were obtained by examining amoeba cultures fixed with 4% paraformaldehyde (PFA) at 4°C for 30 min and stained with 10% SYBR-Gold diluted in dimethyl sulfoxide (DMSO) for 5 min at room temperature. Samples were observed and imaged using a fluorescence microscope (Zeiss Axio Observer 7, Germany).

#### Scanning electron microscope/energy dispersive X-ray spectroscopy (SEM/EDX) analysis

Since amoeba shells can be destroyed during the fixation processing [41], fixation for SEM and EDX analysis was avoided. Instead, amoebae-containing colonies for SEM analysis were placed on 3 μm polycarbonate membrane filters, flash frozen in liquid nitrogen, and then subjected to complete freeze-drying to remove moisture. The filters were scanned, and photomicrographs were obtained using a Phenom Pro desktop SEM (Thermo Fisher Scientific, USA) at an acceleration voltage of 10 kV. To determine shell composition, amoebae grown with dead IMS101 cultures (without obvious trichomes) were collected on filters and air-dried for SEM–EDX analysis (see Supplementary 1.6).

### Co-culturing of amoeba with IMS101

To explore interactions between amoeba and IMS101, a series of laboratory experiments were conducted with amoeba and IMS101 cultures in YBC II medium under the same conditions as those used for amoeba culturing. Some examined only amoeba state and growth, while others simultaneously monitored both amoeba and IMS101. Growth of individual amoeba amended with 1 mL IMS101 supernatant (0.2 μm-filtered) or IMS101 cultures at different growth phases (exponential or declining) were tested in 48-well plates by observing and counting the amoebae directly using an inverted microscope (Nikon Eclipse Ti-S) (Table S1). Simultaneous growth was tested in 250-mL polycarbonate bottles (Nalgene, USA) with the addition of amoebae to IMS101 cultures, both initially in the exponential growth phase, using starting ratios comparable to natural colonies (~10 amoebae/100 IMS101 filaments, Table S1). Here amoebae were sub-sampled after homogenization by shaking, preserved with 2% v/v Lugol’s solution and counted. IMS101 growth was evaluated from Chl*a* measurements (see Supplementary 1.7). Growth rates of IMS101 and amoeba were determined via linear regressions of the natural logarithm of Chl*a* concentrations and cell numbers versus time, respectively.

## Results

### Occurrences of amoebae within Red Sea *Trichodesmium* colonies

**Figure 1:**
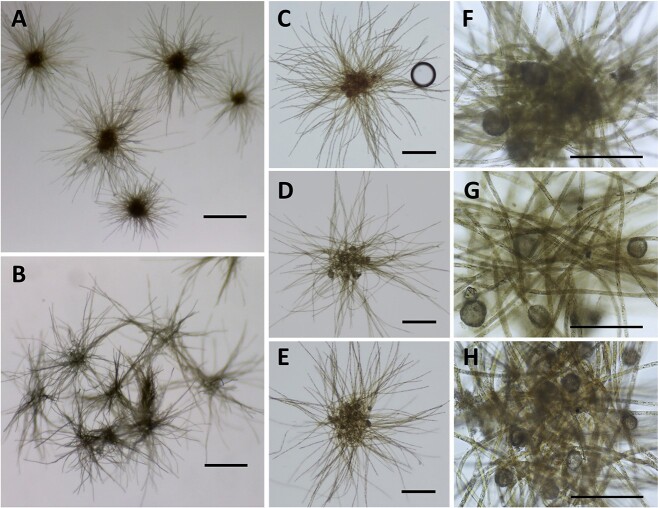
**Red Sea puff *Trichodesmium* colonies associated with the amoeba *Trichosphaerium micrum*.** Fresh colonies were collected from the Gulf of Aqaba on a daily basis during spring seasons of 2021 and 2022, examined and imaged with a stereoscope and microscope. **(A, B).** Stereoscopic images of typical amoebae-containing colonies (A) and amoebae-free colonies (B), scale bars are 500 μm. **(C, D, E).** Microscopic images of representative colonies with *Trichosphaerium micrum* taken at 4× objective lens, scale bars are 200 μm. **(F, G, H).** Close up of the colony’s core, taken at 20× objective lens, scale bars are 100 μm.

**Figure 2:**
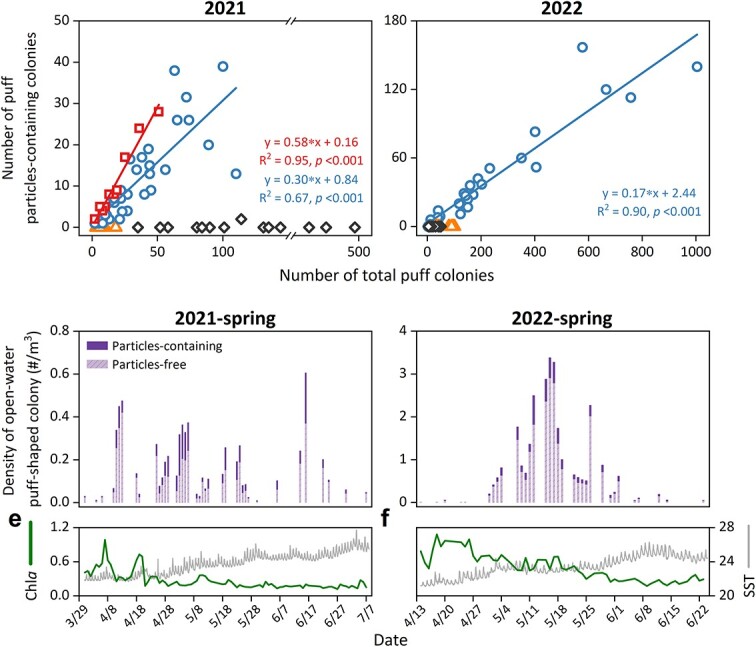
**Occurrences of *Trichosphaerium micrum* within different *Trichodesmium* colony morphotypes in the Red Sea, collected in nearshore or open-water zones during 2021 and 2022.** Daily and seasonal data are presented as number of colonies (A, B) and as colony density in the open water (C, D). **(A, B).** Correlations between the total number of puff or tuft *Trichodesmium* colonies and those associated with the amoeba *Trichosphaerium micrum* collected in nearshore (NS) or open-water (OW) zones in spring and autumn of 2021 (A) and 2022 (B). Pearson’s correlation analysis was used to assess the correlations. The fraction of amoebae-containing colonies in NS and OW zones on the same sampling day is shown in Fig. S4. **(C, D).** Temporal variations in the density of puff *Trichodesmium* colonies in OW zone, showing amoebae-free colonies (Particles-free) and amoebae-containing colonies (Particles-containing). **(E, F).** Sea surface temperature (SST, thin and grey lines) and Chl*a* concentration (thick and green lines) in the Gulf of Aqaba water.

Throughout several intensive studies characterized by the frequent collection of natural *Trichodesmium* colonies from the Gulf of Aqaba in the Red Sea, unusual puff colonies with spherical, deep brown and dense cores were documented (Fig. 1A). These colonies were distinct from the typical puff colonies dominated by *Trichodesmium thiebautii* [42], characterized by looser trichome assemblies and lighter-colored cores (Fig. 1B). Examination of these colonies with higher microscopic magnifications revealed abundant (ranging from 10–40 particles per colony), identically-sized large (average diameter of 32 ± 5 μm) spherical brown particles (Fig. 1C–H, Fig. S3). The occurrence of these particles within colonies was examined through detailed field surveys conducted during four *Trichodesmium* bloom seasons in spring and autumn of 2021 and 2022. Over 10 000 puff colonies and 500 tuft-shaped colonies were collected from nearshore and/or open-water zones over 100 separate days and examined for the presence of these particles, which were later identified as the testate amoeba *Trichosphaerium micrum* (see later). The surveys revealed a distinct seasonal pattern where during both spring seasons, amoebae were detected on most sampling days, while in autumn, they were rarely detected (Fig. 2). The number of puff colonies associated with amoebae varied daily (Fig. 2C and D), but was positively correlated with the total colony number throughout the spring of 2021 (r^2^ = 0.67) and 2022 (r^2^ = 0.90) (Fig. 2A and B), indicating a robust and recurrent annual association. In 2021, colonies were collected from coastal (IUI Pier) and off-shore (open-water) locations. Notably, the fraction of amoebae-containing colonies was significantly higher in the coastal samples (58%) compared to the open-water samples (32%), with a *p* value of <0.001 (Fig. S4). In the open-water, the fraction of amoebae-containing colonies was higher in spring 2021 (32%, n = 39 days) compared to spring 2022 (17%, n = 33 days) (Fig. 2A and B). The lower amoebae occurrence in spring 2022 coincides with higher numbers of *Trichodesmium* colonies (0–4 colonies/m^3^) (Fig. 2C and D). In the survey, over 500 tuft *Trichodesmium* colonies and some other unidentified puff colonies were also examined*.* Tuft colonies typically occurred at lower abundance (<0.1 colonies/m^3^). Neither tuft nor unidentified puff colonies were found to be associated with the same amoebae (Fig. 2A and B).

### Obtaining amoebae cultures from Red Sea *Trichodesmium* colonies

**Figure 3:**
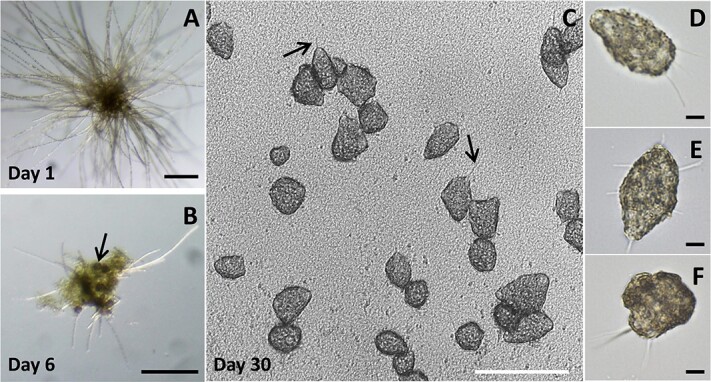
**State changes of *Trichodesmium* spp. or *Trichosphaerium micrum* in cultures of individual amoebae-containing *Trichodesmium* colonies. (A).** Image of a typical amoebae-containing colony after 1-day incubation in a well of a 48-plate containing 1 mL FSW, scale bar = 200 μm. More details of 1-day incubation are shown in Fig. S2. **(B).** Image of the colony detritus clump associated with spherical *Trichosphaerium micrum* (arrow) after a 6-day incubation, scale bar = 200 μm. (C). After a 30-day incubation, an inverted microscope image of flat *Trichosphaerium micrum* with pseudopodia (arrows) on the well bottom, scale bar = 100 μm. (D-F). Upright light microscope images of representative *Trichosphaerium micrum* picked from the well in (C), scale bars = 10 μm.

Amoeba cultures were established by incubating ~50 amoebae-containing colonies separately in 48-well plates with FSW. Over several months the wells were closely monitored with regards to *Trichodesmium’s* condition, the amoebae’s morphology and density, and the presence of other eukaryotes (Table S1). Within 1–2 weeks, *Trichodesmium* died and disintegrated from the wells but the amoebae remained and gradually started transforming (Fig. 3). The spherical dark brown spheres became flat and elongated and took diverse shapes and some started moving along the bottom of the plate wells, exhibiting typical amoeboid locomotion by extruding and retracting pseudopodia (Fig. 3C–F) [26]. In the next few weeks, amoebae were multiplying, and their cell numbers increased (Fig. 3C). After three months, amoebae numbers increased in 85% of the wells, reaching densities of hundreds to thousands per well. Amoebae were not found in the 20 control wells incubated with amoebae-free colonies. Interestingly, other eukaryotes, such as diatoms or dinoflagellates also grew in addition to amoebae, suggesting these colonies are engaged in multiple associations with eukaryotes. The successful culturing attempts suggest that the amoebae benefit from decaying colonies*,* possibly by feeding on their associated microorganisms or by digesting organic matter derived from the colony. Parallel attempts to obtain amoebae culture in the absence of colonies failed, even when using freshly collected seawater containing bacteria or phytoplankton prey.

### Characterization and identification of the associated amoebae

Attempts to amplify the amoeba 18S rRNA gene from natural amoebae, whether associated with, or separated from, *Trichodesmium* colonies yielded irreproducible results due to contamination by other associated eukaryotes, such as copepods. This challenge was successfully addressed by using two actively growing amoeba cultures (Amoe_Colony1 and Amoe_Colony2, Table S2), originating from two individual *Trichodesmium* colonies and free of other eukaryotes. The resulting six 18S rRNA gene sequences (~2430 bp; accession numbers OR601703-OR601708) had the highest similarity to the amoeba *Trichosphaerium* sp. ATCC 40318 (EU273464–71; 70–75% coverage and 90% identity). Putative *Trichosphaerium* Amoe_Colony1 and Amoe_Colony2 sequence identities ranged between 96% and 98% (Table S2), variation that is consistent with 18S rRNA gene polymorphism in the *Trichosphaerium* [43].

**Figure 4:**
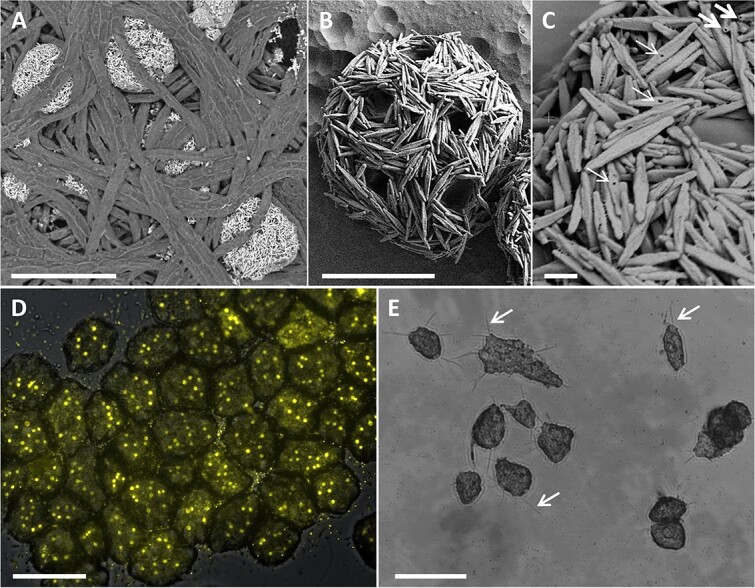
**Cell surface (A-C), nuclei (D) and pseudopodia (G) of amoeba *Trichosphaerium micrum* associated with puff *Trichodesmium* colonies. (A).** Scanning electron microscopic (SEM) image of *Trichosphaerium micrum* residing within the colony core. **(B).** Horizontally arranged spicules divided by circular apertures, scale bar = 10 μm. **(C).** The spicules have opened ends (thick arrows) and a hole on the midpoint of one side (thin arrows), scale bar =1 μm. **(D).** Gold staining of multiple nuclei (yellow spots). **(E).** Flat *Trichosphaerium micrum* with extended pseudopodia (arrows), attaching to the bottom of the 48-plate. *Trichosphaerium micrum* in (b-e) was co-cultured with *Trichodesmium erythraeum* (strain IMS101) in the laboratory. Scale bars in (A, D, E) are 50 μm.

SEM imaging revealed morphological characteristics typical of the genus *Trichosphaerium*, with cells covered by dense spicules (Fig. 4A–C and Table S3). SEM-XRD analysis confirmed that the spicules are made of calcite, similar to previous studies [44]. Further morphological features (Figs. 3 and 4) and comparisons to studies detailing the morphology of *Trichosphaerium* species [10, 44, 45], enabled categorization at the species level as *Trichosphaerium micrum.* The most discriminative feature supporting this identification is the resemblance among the spicules, which are elongated (1–3 μm), blunt-ended, and hollow, each having a hole in their center on one side (Fig. 4C and Table S3). The spicules are arranged horizontally in multiple layers, forming a flexible shell with clear openings, probably for pseudopodia (Fig. 4B and C and Table S3) [44]. Other features that show similarities are size (32 vs. 36 μm of average cell diameter) (Fig. S3A), the number of granular nuclei per cell (10 vs. 9 nuclei/cell) (Fig. 4D), and locomotion by hair-like pseudopodia (dactylopodia) (Fig. 3C–F and Fig. 4E). Similar morphological features were identified for amoebae collected throughout the annual time-series, suggesting that these amoebae likely belong to the same species, *Trichosphaerium micrum*, and that its association with *Trichodesmium* colonies is likely a regular phenomenon in the Red Sea.

### Untangling *Trichodesmium - Trichosphaerium* associations - co-culturing studies

**Figure 5:**
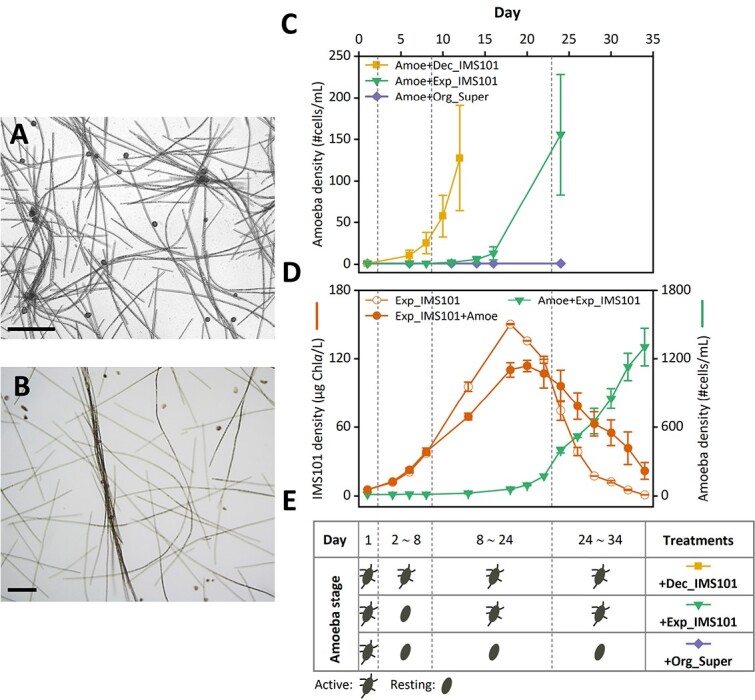
**Growth and state transitions of *Trichosphaerium micrum* and *T. erythraeum* in co-cultures with varied volumes. (A, B).** Upright light microscope (A) and inverted microscope (B) images of actively growing *Trichosphaerium micrum* with stationary phase *T. erythraeum* (strain IMS101) on the day 15–24 of co-cultures in (C, green triangles). Scale bars are 200 μm. **(C).** Growth curves of individual *Trichosphaerium micrum* cultured in wells of 48-plates initially containing 1 mL exponential (+Exp_IMS101, green triangles, n = 9) or declining (+Dec_IMS101, yellow squares, n = 6) IMS101 cultures, or organics-containing supernatant from declining IMS101 cultures (with both IMS101 and its associated bacteria removed by 0.22 μm filtration) (+Org_Super, purple diamonds, n = 8). **(D).** Growth curves of co-cultured *Trichosphaerium micrum* (Amoe+Exp_IMS101, green triangles) and IMS101 (Exp_IMS101 + Amoe, solid orange circles), and monocultured IMS101 (Exp_IMS101, open orange circles) in 200 mL YBC II. The initial phase of IMS101 is in exponential phase. Each treatment was performed in biological duplicates. (E). Summary of *Trichosphaerium micrum* state transitions corresponding to conditions in (C, D). *Trichosphaerium micrum* was from the sequenced culture Amoe_Colony1 (Table S2). Error bars represent the standard deviation (SD) of biological samples.

Striving to understand the interactions between the two members of the association, several co-culturing experiments with a laboratory-grown strain of *T. erythraeum* (IMS101) and newly isolated *Trichosphaerium micrum* were conducted. *Trichosphaerium micrum’s* growth was monitored when amended with IMS101 cultures in different growth phases (Table S1 and Fig. 5). *Trichosphaerium micrum* remained active and grew exponentially when mixed with declining “old” IMS101, establishing a growth rate of 0.43 ± 0.05 d^−1^ (Fig. 5C and E, yellow squares). However, when amended with exponentially growing “young” IMS101 cultures, *Trichosphaerium micrum* entered a resting state and did not grow for the first ten days (Fig. 5C and E, green triangles). Later, as IMS101 cultures started to enter stationary phase, *Trichosphaerium micrum* became active again (Fig. 5A and B), growing at a rate of 0.37 ± 0.04 d^−1^ (Fig. 5C and E, green triangles). Observations showed *Trichosphaerium micrum* can attach to floating IMS101 filaments, though the majority generally attached to the bottom of culture vessels (Fig. 5B). No microscopic evidence for IMS101 engulfment by *Trichosphaerium micrum* was found, suggesting that *Trichosphaerium micrum* is consuming bacteria and/or organic matter, both of which are more abundant in late stationary phase “old” IMS101 [39]. Organic matter was ruled out as a primary food source, as the organic-containing supernatant from declining IMS101 cultures, with both IMS101 and its associated bacteria removed by 0.22 μm filtration, did not support *Trichosphaerium micrum* growth (Fig. 5C and E, purple diamonds).

Testing further mutual interactions, a full growth cycle was obtained for different treatments (Fig. 5D). As in the previous co-culturing experiment (Fig. 5C), *Trichosphaerium micrum* did not grow during the first 10 days when IMS101 was still in exponential phase, but then became active and reached a maximum growth rate (Fig. 5D and E). IMS101 growth rates were unaffected by *Trichosphaerium micrum* in the first 10 days of exponential growth (Fig. 5D, orange lines with solid *vs.* open circles). In the next 10 days *Trichosphaerium micrum* had a mild negative impact on IMS101 that grew slower and obtained lower maximal biomass compared to the control. In the last stage of the experiment (>24 days), slower decline of IMS101 was observed in the presence of *Trichosphaerium micrum* (Fig. 5D).

### Effect of *Trichosphaerium micrum* on bacterial community of puff *Trichodesmium* colonies

**Figure 6:**
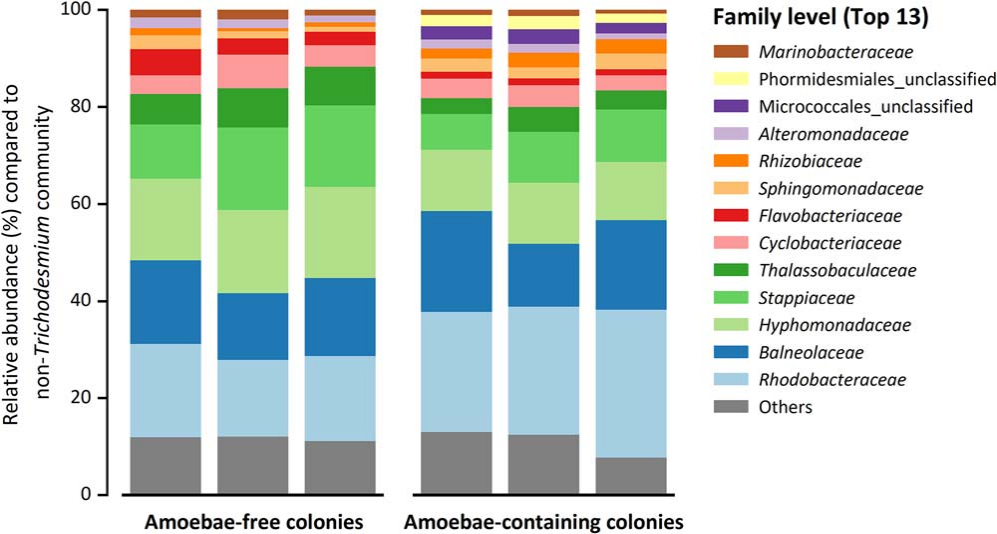
**The effect of *Trichosphaerium micrum* on bacterial compositions in puff *Trichodesmium* colonies.** Barplot showing the relative abundance of the top 13 epibionts at the family taxonomy level in amoebae-containing and amoebae-free colonies, with a mean relative abundance >1% of the epibiont ASVs.

Analysis of field-collected puff colony 16S rRNA gene (V4 region) sequences demonstrated that *Trichodesmium* spp. accounted for 40–55% of 16S rRNA genes sequences across all samples, while the remaining 16S rRNA sequences (45–60%) were from *Trichodesmium* microbiome (Fig. S5). The samples with and without amoebae had distinct patterns in relative community composition (Fig. 6), although these differences were not significant either with *Trichodesmium* (unweighted UniFrac, *p* = 0.09, Fig. S5A) or without (excluding *Trichodesmium* ASVs unweighted UniFrac, *p* = 0.10, Fig. S5B). The presence of *Trichosphaerium micrum* did coincide with the presence of several taxonomic groups, including Micrococcales_unclassified and Phormidesmiales_unclassified, with an average relative abundance compared to the non-*Trichodesmium* community of 2.6% and 2.3%, respectively (Fig. 6). The relatively minor effect of *Trichosphaerium micrum* on the colony bacterial community may reflect a lack of active grazing on bacteria by amoebae in resting stages at the time of sampling.

### Effect of calcifying amoebae on the colony buoyancy

**Figure 7:**
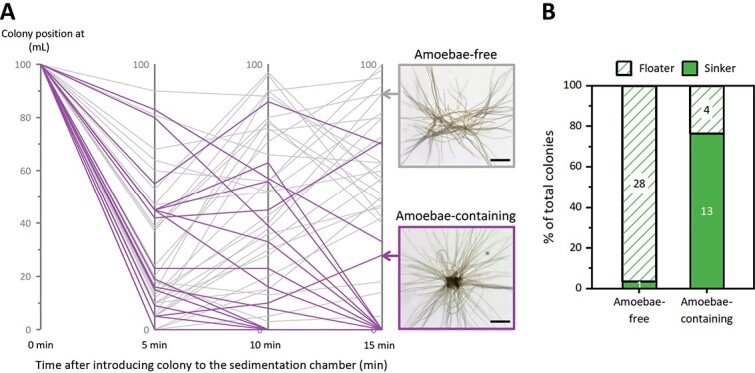
**The effect of *Trichosphaerium micrum* on the buoyancy of puff *Trichodesmium* colonies. (A).** Parallel-coordinates plot illustrating the variations in vertical positions of puff *Trichodesmium* colonies during the sinking experiments. Vertical positions of individual colonies with amoebae (thick and purple lines, n = 17) and without amoebae (thin and grey lines, n = 30) were recorded at 5, 10, and 15 min after placement in the chambers. The vertical positions are measured in mL, representing the distance from the chamber’s water depth. Colony scale bars are 200 μm. **(B).** Summary of the fraction of floaters and sinkers 15 min after placement in the chambers. Colonies that sank to the bottom of the chamber by 15 min are classified as “sinkers,” and those floating in the chamber as “floaters” [32].

Field-collected *Trichosphaerium*-containing *Trichodesmium* colonies were observed at the bottom of the Petri dish within an hour after collection, hinting at a potential adverse impact of this association on colony buoyancy. Sinking experiments showed that both amoebae-containing and amoebae-free colonies were capable of moving up and down in the water chamber (Fig. 7A), but within 15 min the majority (76%) of the amoebae-containing colonies sank to the bottom of the chamber, while only 3% of the amoebae-free colonies did so (Fig. 7B). Based on cell counts and average cell weight, testate, resting stage *Trichosphaerium micrum,* as observed in the field, was calculated to contribute 0.1–1.3 μg to the total weight of 0.4–2.5 μg of trichomes in puff *Trichodesmium* colonies, accounting for 4%–76% of the total colony weight (Table S4 and Fig. S3). Furthermore, the estimated cell density of amoebae-containing colonies ranged from 950–1570 kg/m^3^ (Table S4). Based on the equation we recently published for dust-loaded colonies [32] and considering the range of total amoebae weight per colony, the sinking speed for amoebae-containing colonies was estimated to range from 5 to 51 m·d^−1^, which is faster than that of particle-free colonies (0–9 m·d^−1^) in the previous study [32]. This is consistent with the idea that the added weight from amoebae can accelerate sinking rates of colonies.

## Discussion

Microbial interactions, both in the water and when cells are attached to substrates such as particles, are increasingly recognized as important to the cycling and export of carbon and nitrogen in marine systems [46], and impact the role these resources play in ecosystem dynamics. These interactions can be finely tuned, and in the case of *Trichodesmium* colonies, the heterotrophic bacteria that live on the colonies have been shown to play a critical role in *Trichodesmium* physiological ecology and the concomitant fate of fixed carbon and nitrogen [14, 17]. While these bacterial interactions are important, the role of heterotrophic microeukaryotes that have also been observed in field populations of *Trichodesmium,* has been largely ignored [10, 11, 30]. Through a two-year survey involving over 10 000 *Trichodesmium* colonies, along with searches of metagenome datasets from *Trichodesmium* colonies sampled in 2019 (Table S5), our study documented a novel association among *Trichodesmium* and testate amoebae in the Red Sea recurring annually over three years. The association was predominantly observed in puff *Trichodesmium* colonies and not in tuft *Trichodesmium* or other unidentified puff colonies, suggesting that factors beyond sediment resuspension may facilitate the specific association with *Trichodesmium* puffs. Microscopic observations showed similar morphology of the amoebae collected over the course of the study, and further morphological identification and 18S rRNA gene sequencing suggested that these amoebae likely belong to the same species, *Trichosphaerium micrum*.

As a pivotal consumer, *Trichosphaerium* can feed on bacteria as its sole food source [47, 48], while it has also been reported to prey on microeukaryotes such as diatoms [45], and efficiently digests a wide range of macroalgae [49, 50]. Consequently, *Trichosphaerium* can play a crucial role in microbial food webs and carbon cycling within marine ecosystems [48, 50]. Despite its ecological importance, research on this organism remains scarce, with only about 10 publications spanning from 1899 [51] to the present. The scarcity of research on *Trichosphaerium* is primarily attributed to its polymorphism and complex life-history dynamics (Fig. S6), presenting challenges for study [48, 51]. Our findings, highlighting the recurrent presence of nearshore benthic *Trichosphaerium* in open-water *Trichodesmium* colonies, broaden the documented habitats of *Trichosphaerium*.

The genus *Trichosphaerium*, a widespread benthic amoeba, typically attaches to substrates in nearshore environments [44, 45, 52]. *Trichosphaerium* was observed here in a heavy testate form [51], where CaCO_3_ spicules are synthesized to construct a flexible cell covering [44]. Physical perturbations, such as currents, dislodge testate amoebae from benthic substrates, temporarily suspending them in the water column [53, 54]. This process increases the potential of physical contact between benthic *Trichosphaerium* and floating *Trichodesmium* colonies, potentially facilitating the formation of the association observed here. To mimic such perturbations, we performed experiments on a shaker and indeed observed that *Trichosphaerium* cultures required this physical forcing to associate with natural colonies (Fig. S7). In contrast, under static conditions, even in small volumes (500 μL), no associations were detected. This suggests that physical perturbations are an important factor driving the association. Nearshore colonies positioned closer to the benthic areas where *Trichosphaerium* thrives, confront stronger physical disturbances such as internal and breaking waves [55], compared to those in open water. This increases the likelihood of encountering suspended *Trichosphaerium* and forming associations, partly elucidating the significantly higher frequency of the association observed in nearshore colonies. Other factors, such as amoebae activity [54] and life cycle transitions [51], can influence the abundance of the testate form in the water column. These factors may contribute to the observed seasonality in the distribution of *Trichosphaerium – Trichodesmium* association*,* but further studies of *Trichosphaerium*’s physiological ecology is warranted to more fully resolve this.

Physical perturbations are essential for the passive dispersal of benthic testate amoebae [53]. However, as the dispersal range is generally limited, associating with positively buoyant colonies could prolong *Trichosphaerium*’s retention in the water column, aiding its dispersal between areas and the exploitation of new habitats. Indeed, some shallow benthic organisms traverse areas by attaching to floating organisms [12]. Similarly, many benthic ciliates, dinoflagellates and copepods were reported to take advantage of the positive buoyancy of *Trichodesmium* colonies to exploit pelagic habitats [10, 12, 56].

Exploring the relationship between *Trichosphaerium* and *Trichodesmium* is crucial to defining the nature of their association. By establishing laboratory co-cultures, and experimenting with field-collected colonies, we could simultaneously determine the growth and activities of both *Trichosphaerium* and *Trichodesmium*, and further deduce their interactions in natural colonies. The feeding and growth of amoebae can rapidly adjust based on environmental conditions, such as the abundance of food [54, 57]. Our study suggests that *Trichosphaerium* may commonly be in a low-activity dormant state in healthy colonies, as *Trichosphaerium* exhibited round shapes without pseudopodia extensions in freshly collected colonies, and in co-cultures where *Trichodesmium* was in exponential growth. As colonies age and bacterial abundance increases [10], dormant *Trichosphaerium* may transition into an active state where cells are feeding. While we did not directly observe the presence of pseudopodia in *Trichosphaerium* from *Trichodesmium* field populations, incubations of individual field-collected *Trichodesmium* colonies demonstrated that the majority of *Trichosphaerium* (>85%) could gradually transition from a dormant to active state during colony decay during incubation, confirming dormancy versus active state transitions linked to *Trichodesmium* host physiology. The lack of active *Trichosphaerium* engulfing *Trichodesmium* during these observations and in co-culture experiments suggests that their relationship may not involve direct predation on *Trichodesmium*, even during colony decline, but rather reflect *Trichosphaerium* feeding on the *Trichodesmium* heterotrophic microbiome. This is further supported by the heterotrophic microbiome differences observed between *Trichodesmium* puff colonies collected with and without amoebae, but warrants further study to capture colony decline in the field.

The association between eukaryotes and *Trichodesmium* colonies is typically assumed to be temporary and unstable. For instance, protozoans seem to freely inhabit and leave colonies [12]. In our experiments, we noticed that colonies containing *Trichosphaerium* took a longer vortexing period to open completely into free trichomes compared to those without *Trichosphaerium.* Additionally, they more often stayed in the colony morphology rather than opening to free trichomes during 24-h incubations (Fig. S2C). *Trichosphaerium* is likely to be “trapped” in the puff colony cores, and its presence may contribute to maintaining colony morphology or even serve a role in colony formation from free trichomes (Fig. S2). Finally, the decaying *Trichosphaerium*-containing colonies tended to form large organic clumps with the amoeba buried within them. These observations suggest a physically tight association between *Trichosphaerium* and puff colonies, not easily disrupted by physical perturbations or the health condition of colonies.

*Trichosphaerium* CaCO_3_ biomineralization has the potential to form limestone mats, and contribute to the marine carbonate cycle [52], in systems where it occurs. Positive attachment and the secretion of extracellular mucin [52] to “glue” the CaCO_3_ spicules enclosing *Trichosphaerium* could also play a role in “gluing” colonies and maintaining the association. This tight association could ensure that *Trichosphaerium* remains within colonies even during changes in *Trichodesmium* physiological ecology. The presence of calcifying *Trichosphaerium* had a significant effect on the buoyancy of colonies as demonstrated by the sinking experiments. *Trichodesmium* buoyancy is typically thought to be modulated by the physiology of its cells [58, 59], and colony buoyancy among other factors is thought to minimize the flux of *Trichodesmium* nitrogen and carbon export [60]. The tight physical association with *Trichosphaerium* and ballasting by CaCO_3_ spicules may enhance the downward export of these open water colonies into the deep ocean, thus re-shaping the flux of new nitrogen from supporting primary production in nitrogen-limited surface waters to exporting particulate nitrogen to deep waters. Several recent studies have noted the presence of *Trichodesmium* in sediment traps, even from depths as great as 1000 m [8, 9], which has renewed a focus on the factors that modulate diazotroph export processes. If the patterns observed here hold true across other systems, this association provides a mechanism for driving *Trichodesmium* carbon and nitrogen export.

## Conclusion

Untangling the myriad of microbial interactions underpinning marine ecosystem structure and function is an ongoing challenge in microbial ecology. Here we identified a novel recurrent association between a testate amoeba and the cyanobacterial diazotroph *Trichodesmium*. In this association, *Trichosphaerium* appears to feed on the *Trichodesmium* microbiome*,* which re-shapes microbiome community composition and thus may impact *Trichodesmium*–microbiome interactions. Further the *Trichosphaerium* can enhance colony sinking rates as well as the flux of newly fixed carbon and nitrogen. Taken together, these interactions add complexity to predicting *Trichodesmium* physiological ecology, including the role this association could play in reshaping the flux and fate of carbon and nitrogen in marine ecosystems.

*Trichosphaerium* has been reported in other coastal oceans [44] that frequently experience *Trichodesmium* blooms, such as Fiji, the Great Barrier Beef, and the Gulf of Mexico [61–65]. Examination of published images suggests that colonies collected from several sites exhibit morphologies similar to the *Trichosphaerium*-containing colonies in the Red Sea, with a dense colony core containing visible round particles (Fig. S8). Given the global significance of *Trichodesmium*, this association may be a new critical facet of the microbial interactions that underpin the fixation and fate of carbon and nitrogen in tropical and subtropical systems. This association likely occurs in other areas, and needs to be considered in the context of predicting how microbial interactions influence carbon and nitrogen biogeochemistry.

## Supplementary Material

Amoebae_Tricho_Colony-suppl_ycae137

## Data Availability

The six *Trichosphaerium micrum* 18S rRNA gene sequences are deposited in the NCBI GenBank (OR601703-OR601708). The 16S rRNA gene amplicon sequences are deposited under BioProject ID number PRJNA1020796.
