# Supplementary material for: Recurrent association between Trichodesmium colonies and calcifying amoebae
Source: ISME Commun. 2024 Nov 4;4(1):ycae137. doi: 10.1093/ismeco/ycae137 (PMC11575492; doi:10.1093/ismeco/ycae137)
Supplement: Amoebae_Tricho_Colony-suppl_ycae137 [file amoebae_tricho_colony-suppl_ycae137.pdf]

Supplementary Materials for

Recurrent association between *Trichodesmium* colonies and calcifying amoebae

Futing Zhang[1,2*], Siyuan Wang[1,2], Anna-Neva Visser[1,2,7], Coco Koedooder[1,2,3], Meri Eichner[4], O. Roger Anderson[5], Sonya T. Dyhrman[5,6] and Yeala Shaked[1,2]

[1]The Fredy and Nadine Herrmann Institute of Earth Sciences, Hebrew University of Jerusalem, The Edmond J. Safra Campus, Jerusalem 9190401, Israel

[2]The Interuniversity Institute for Marine Sciences in Eilat, Coral Beach P.O.B 469, Eilat 8810302, Israel

[3]Israel Limnology and Oceanography Research, Tel-Shikmona P.O.B 8030, Haifa 3108000, Israel

[4]CentreAlgatech, Institute of Microbiology of the Czech Academy of Sciences, Novohradská 237, Třeboň 37901, Czech Republic

[5]Biology and Paleo Environment, Lamont-Doherty Earth Observatory of Columbia University, 61 Route 9W, Palisades, NY 10964, United States

[6]Department of Earth and Environmental Sciences, Columbia University, 1200 Amsterdam Avenue, New York, NY 10027, United States

[7]Present address: Friedrich-Alexander-University Erlangen-Nuernberg (FAU), Department Geographie und Geowissenschaften, Erlangen 91054, GeoZentrum Nordbayern, Schlossgarten 5, Germany

* Correspondence: Futing Zhang (futing.zhang@mail.huji.ac.il)

This PDF file includes:

1. Materials and Methods
2. Tables S1 to S5
3. Figures S1 to S8
4. References

1. Materials and Methods

1.1 Enumeration of amoeba *Trichosphaerium micrum* in individual puff-shaped *Trichodesmium* colonies

While the presence of amoebae within live colonies was observed clearly under the microscope (Fig. 1), enumerating them within the compacted cores was not feasible. Attempts to count amoebae by opening colonies, similar to the method used for counting *Trichodesmium* trichomes, often resulted in amoebae aggregation or adhesion to the walls of Eppendorf tubes during shaking and vortexing. Due to the mineralized shell of testate *Trichosphaerium micrum* (Fig. 4) [1], they were separated from *Trichodesmium* for counting by soaking individual colonies in double distilled water (DDW) for 2 min (Fig. S1). The residual clump was placed on a 3-µm pore-size polycarbonate membrane filter (Merck Millipore, Germany) and washed gently with DDW. This treatment led to complete disintegration and loss of *Trichodesmium* trichomes, whereas the testate amoebae were retained on the filter. The amoebae were carefully observed and counted under the microscope. In total, 41 amoebae-containing colonies, randomly picked on different years and days, were examined.

1.2 16S rRNA gene amplification and sequencing

Samples of two morphologies, amoebae-containing and amoebae-free *Trichodesmium* colonies, were separately collected in biological triplicates (30 colonies of each) on different days during spring 2021 by hand-picking under the microscopy. Samples were gently vacuum-filtered on 0.2-µm Supor filters (Pall Life Sciences, USA). The filters were flash frozen in liquid nitrogen and stored at -80°C until DNA extraction. Total DNA was extracted using the QIAamp UCP DNA Micro Kit (Qiagen, Germany) according to the manufacturer’s instructions, with an initial bead-beating step. Regions (16S rRNA V4) were amplified using the specific primers, 515F (5’-GTGCCAGCMGCCGCGGTAA-3’) and 806R (5’-GGACTACHVGGGTWTCTAAT-3’), with adapters and barcodes. Sequencing libraries were generated using TruSeq DNA PCR-Free Sample Preparation Kit (Illumina, USA) following manufacturer's recommendations and index codes were added. The library quality was assessed on the Qubit 4.0 Fluorometer (Thermo Fisher Scientific, USA) and Agilent Bioanalyzer 2100 system. The library was sequenced on an

IlluminaHiSeq2500 platform and 250 bp paired-end reads were generated at Hylabs (Jerusalem, Israel).

1.3 Physical separation of amoeba *Trichosphaerium micrum* from puff-shaped *Trichodesmium* colonies

To separate amoebae, 30 amoebae-containing colonies were thoroughly opened by shaking and vortexing, and the mixture was transferred to a Petri dish. Amoebae sank and stuck to the bottom of the Petri dish within a few minutes, while *Trichodesmium* trichomes remained floating. The trichomes were then removed by gently pipetting, and the amoebae were washed by resuspending three times in FSW. Clean separated amoebae (1-5 each) were incubated with 1 mL not-filtered seawater that contained naturally-occurring bacteria and microeukaryotes.

1.4 DNA extraction, 18S rRNA gene amplification and sequencing of amoeba *Trichosphaerium micrum*

Two independent amoebae cultures Amoe_Colony1 and Amoe_Colony2 (Table S2), originating from two Red Sea puff-shaped *Trichodesmium* colonies, were used for amoeba DNA extraction. Total DNA was extracted using the QIAamp UCP DNA Micro Kit (Qiagen, Germany) according to manufacturer instructions. PCR reactions were: an initial denaturation (95°C for 10 min), followed by 39 cycles of denaturation at 95°C for 30 s, annealing at 50°C for 1 min and extension at72°C for 2 min, and a final extension at 72°C for 10 min. All reactions were carried out on a LabCycler (SensoQuest GmbH, Germany). Nuclease-free water and total DNA extracted from amoebae-free colonies were used for negative controls. PCR products were separated on 1.2% agarose gels, and unique DNA products were purified using QIAquick Gel Extraction Kit (Qiagen) following the manufacturer instructions. The purified PCR products were then inserted into pCR™4-TOPO® vectors (Invitrogen, USA), and transformed into TOP10 competent cells. Plasmid DNA from six positive clones was separately extracted using the PureLink™ Quick Plasmid Miniprep Kit (Invitrogen, USA) and sequenced using primers: M13-F(-20) (5’-GTAAAACGACGGCCAG-3’), M13-R (5’-CAGGAAACAGCTATGAC-3’), A18-F (5’-GGTAGGGTAACGGCCTAC-3’) and A18-R (5’-CAGGAACCCGCTGAACTAAG-3’).

1.5 Estimation of average *Trichosphaerium micrum* cell weight

The average amoeba cell weight was estimated by weighing cells using a microbalance [2]. Amoebae cultured with declining IMS101 (no obvious trichomes) were counted, and their average cell diameter was measured using an inverted microscope. Amoebae cultures (10 mL each) were concentrated on 3-µm pore size polycarbonate membrane filters. Each filter contained >5,000 amoeba cells. In order to minimize the effects of bacteria, organic matter and salts on the weight measurement, the filters were washed five times by flushing through DDW. The filters were then completely dried at room temperature. The average cell weight was calculated based on the net weight and total number of amoebae on a filter. The average cell density was then calculated by dividing the average cell weight by the average cell volume, which was derived from the cell diameter.

1.6 SEM-EDX analysis

Air-dried filters with amoebae were mounted on aluminum stubs using adhesive carbon tape. To increase sample conductivity and enhance imaging performance, a 2 to 3 mm Iridium layer was applied using a sputter coater (Quorum Q150VS plus, UK), reaching a minimum pressure of 6-7 $x10^{-5}$ mbar of Ar. For SEM imaging, a Thermo Apreo 2.0 SEM with a vacuum chamber pressure of ~6 $x10^{-6}$ Torr was used. Image acquisition was performed using the Everhart-Thornley chamber detector (ETD), as well as the Trinity detection system (acceleration voltage of 2 kV, working distances 2 to 5 mm). SEM-EDX analysis was carried out using the Thermo Apreo 2.0 SEM equipped with the Apreo EDX detector. EDX spectra were acquired at an acceleration voltage of 10 kV, a probe current of 1.6 nA, and a working distance of 10 mm. Suitable spots were selected a priori using settings that permitted a higher spatial resolution. Spectra were acquired either from small areas that covered the selected structures (hot spots) or over a transect, including different structures (e.g., spicules, empty spots in between). The acquisition time for each spectrum varied between 27 to 37 seconds.

1.7 Chl*a* measurement of *Trichodesmium erythraeum* IMS101

Since Chlorophyll autofluorescence of amoebae was undetected, the biomass of IMS101 in co-cultures was estimated by measuring Chl*a* concentration without

separating amoebae. The IMS101 culture was collected in the middle of the photoperiod by vacuum filtering on 3-µm pore size polycarbonate membrane filters (Merck Millipore, Germany). The filters placed in Eppendorf tubes containing 90% (vol/vol) methanol were heated at 70°C for 6 min. The filter was then removed and the extract in the tube was centrifuged at 14,000 rpm for 5 min. The absorbances of the supernatant were measured at 665 and 750 nm using a spectrophotometer (Agilent Technologies, Germany) and Chl*a* concentration was calculated based on [3].

2. Tables S1 to S5

Table S1. Overview of detailed incubation experiments involving Red Sea puff-shaped *Trichodesmium* colonies and amoeba *Trichosphaerium micrum* separated from them, as well as the co-culturing of *Trichosphaerium micrum* with the laboratory strain *Trichodesmium erythraeum* IMS101. Experimental conditions, time points, and key observations are provided.

**Goals, respective figures**	**Incubations**	**Initial state of colony or IMS101**	**Biological replicates**	**Initial biomass**
• Observation of colony's health and morphology after a 24-hour incubation, **Fig. S2**	Amoebae-containing colonies	Healthy[a]	53	Single colony in 1 mL FSW
	Amoebae-free colonies	Healthy	20	Single colony in 1 mL FSW
• Obtaining amoebae cultures after 1-3 months incubation, **Fig. 3**	Natural amoebae separated from colonies	NA	~60	1-5 amoebae in 1 mL not-filtered seawater
• Monitoring growth and state transitions of amoeba, **Fig. 5c&e**	Amoebae + IMS101	Exponential	9	Single amoeba with 1 mL IMS101 culture
	Amoebae + IMS101	Decline	6	Single amoeba with 1 mL IMS101 culture
	Amoebae + filtered medium of declining IMS101 culture	NA	8	Single amoeba with 1 mL 0.22 µm filtered media of declining IMS101
• Monitoring growth and state transitions of amoeba and IMS101, **Fig. 5d&e**	Amoebae + IMS101	Exponential	2	~(13 amoebae and 100 trichomes)/mL in 250 mL YBC II media[b]
	IMS101	Exponential	2	~100 trichomes/mL in 250 mL YBC II media

NA: not applicable. **a**: Healthy colonies displayed whole and dark-brown trichomes. **b**: The original biomass ratios of IMS101 to *Trichosphaerium micrum* were determined according to the average ratio of *Trichodesmium* trichomes to amoebae cells in natural colonies.

Table S2. Matrix of sequence identity for amoeba *Trichosphaerium micrum* 18S rRNA sequences. The identity of clones from the different *Trichosphaerium micrum* cultures are similar to those from the same cultures. Two *Trichosphaerium micrum* cultures, Amoe_Colony1 and Amoe_Colony2, were originated from two individual Red Sea puff-shaped *Trichodesmium* colonies incubations .

**Sequences**		**Amoe_Colony1**			**Amoe_Colony2**		
		clone1	clone2	clone3	clone1	clone2	clone3
**Amoe_Colony1**	clone1	1.000	0.969	0.960	0.976	0.969	0.975
	clone2	---	1.000	0.974	0.966	0.961	0.971
	clone3	---	---	1.000	0.953	0.961	0.964
**Amoe_Colony2**	clone1	---	---	---	1.000	0.965	0.967
	clone2	---	---	---	---	1.000	0.961
	clone3	---	---	---	---	---	1.000

Table S3. Spicule structure and orientation of testate *Trichosphaerium* spp. from this and previous studies. Images of *T. micrum*, *T. sieboldin* and *T. platyxyrum* are from (Angell 1975) [1], (Sheehan and Banner 1973) [4], and (Angell 1976) [5], respectively, with permission.

Species and reference	Spicule structure and orientation	Cell surface	Spicules
T. micrum (This Study)	• 1~3 µm in length<br>• Hollow, 3-sided, blunt-ended<br>• A hole in the center of spicules<br>• Lying flat on the cell surface	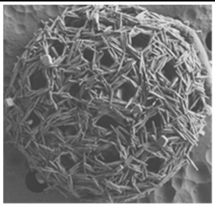	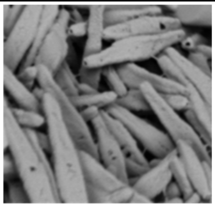
T. micrum (Angell 1975) [1]		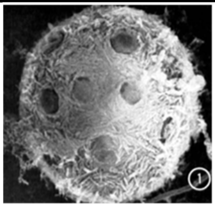	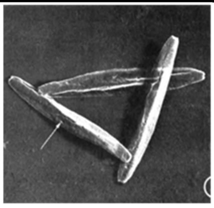
T. sieboldi (Sheehan and Banner 1973) [4]	• Solid, 3-sided, pointed<br>• Perpendicular to the cell surface	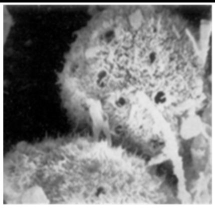	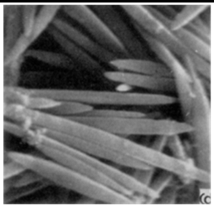
T. platyxyrum (Angell 1976) [5]	• Thin, blade-shaped, beveled or square-ended<br>• Perpendicular to the cell surface	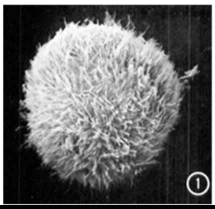	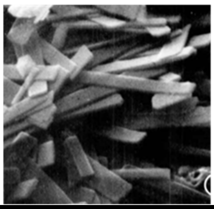

Table S4. Estimation of the cell density range of amoebae-containing puff-shaped *Trichodesmium* colonies in the Red Sea. The range of total *Trichodesmium* spp. cell volume was estimated in our previous study [6] and the accompanying range of total number and weight of associated amoeba *Trichosphaerium micrum* is presented in Fig. S3.

Parameters	Colony Size		
	Small	Middle	Big
Trichodesmium cell density (ρ_{colony}, kg/m^3) [7]	920-1065		
Total *Trichodesmium* cell volume (µm^3/colony) [6]	3.9 x 10^5	1.4 x 10^6	2.3 x 10^6
Total *Trichodesmium* weight (µg/colony)	0.36-0.42	1.2-1.4	2.1-2.5
Range of associated *Trichosphaerium micrum* (#/colony)	4-38		
Average *Trichosphaerium micrum* cell density (ρ_{amoeba}, kg/m^3)	1848		
Average *Trichosphaerium micrum* cell volume (µm^3)	1.9 x 10^4		
Total associated *Trichosphaerium micrum* weight (µg/colony)	0.14-1.3		
Cell density of amoebae-containing colony ($\rho_{colony+amoeba}$, kg/m^3)	1068-1569	968-1334	949-1248

Table S5. BLAST results using the identified 18S rDNA (V4 region) gene of amoeba *Trichosphaerium micrum* to search the metagenome datasets (JGI Proposal ID: *504276*) from *Trichodesmium* colonies sampled in the Gulf of Aqaba in May 2019. High identity scores and low E-values indicate the presence of *Trichosphaerium micrum* 18S rDNA genes in the datasets, suggesting that these amoebae were present in the *Trichodesmium* colonies collected during the spring of 2019.

**Query Sequence Name**	**Scaffold ID**	**Genome ID**	**Metagenome Name**	**E-value**	**Identities**
Trichosphaerium micrum (18S-V4)	Ga0451486_037184	3300044452	Simulated microbial communities of *Trichodesmium* and heterotrophic bacteria from Gulf of Aqaba, Eilat, Israel - Tfinal+54FeOx-3	0	510/545 94%
Trichosphaerium micrum (18S-V4)	Ga0451487_137727	3300044453	Simulated microbial communities of *Trichodesmium* and heterotrophic bacteria from Gulf of Aqaba, Eilat, Israel - Tfinal+54FeOx-2	0	495/548 90%
Trichosphaerium micrum (18S-V4)	Ga0451487_115529	3300044453	Simulated microbial communities of *Trichodesmium* and heterotrophic bacteria from Gulf of Aqaba, Eilat, Israel - Tfinal+54FeOx-2	8.00E-60	126/126 100%
Trichosphaerium micrum (18S-V4)	Ga0451486_048470	3300044452	Simulated microbial communities of *Trichodesmium* and heterotrophic bacteria from Gulf of Aqaba, Eilat, Israel - Tfinal+54FeOx-3	2.00E-50	111/112 99%
Trichosphaerium micrum (18S-V4)	Ga0451487_067339	3300044453	Simulated microbial communities of Trichodesmium and heterotrophic bacteria from Gulf of Aqaba, Eilat, Israel - Tfinal+54FeOx-2	2.00E-50	111/112 99%
Trichosphaerium micrum (18S-V4)	Ga0451482_063831	3300044274	Simulated microbial communities of *Trichodesmium* and heterotrophic bacteria from Gulf of Aqaba, Eilat, Israel - Tfinal-2	1.00E-48	106/106 100%

3. Figures S1 to S8

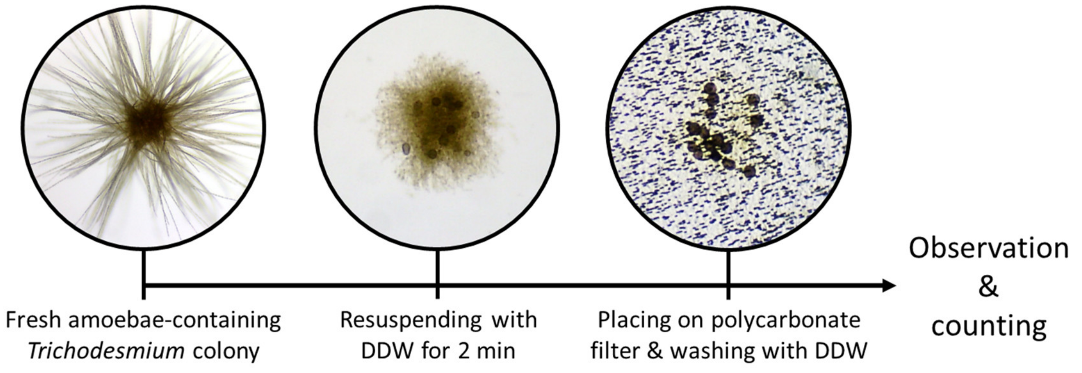

Figure S1. Workflow of counting amoeba *Trichosphaerium micrum* embedded in the cores of Red Sea puff *Trichodesmium* colonies.

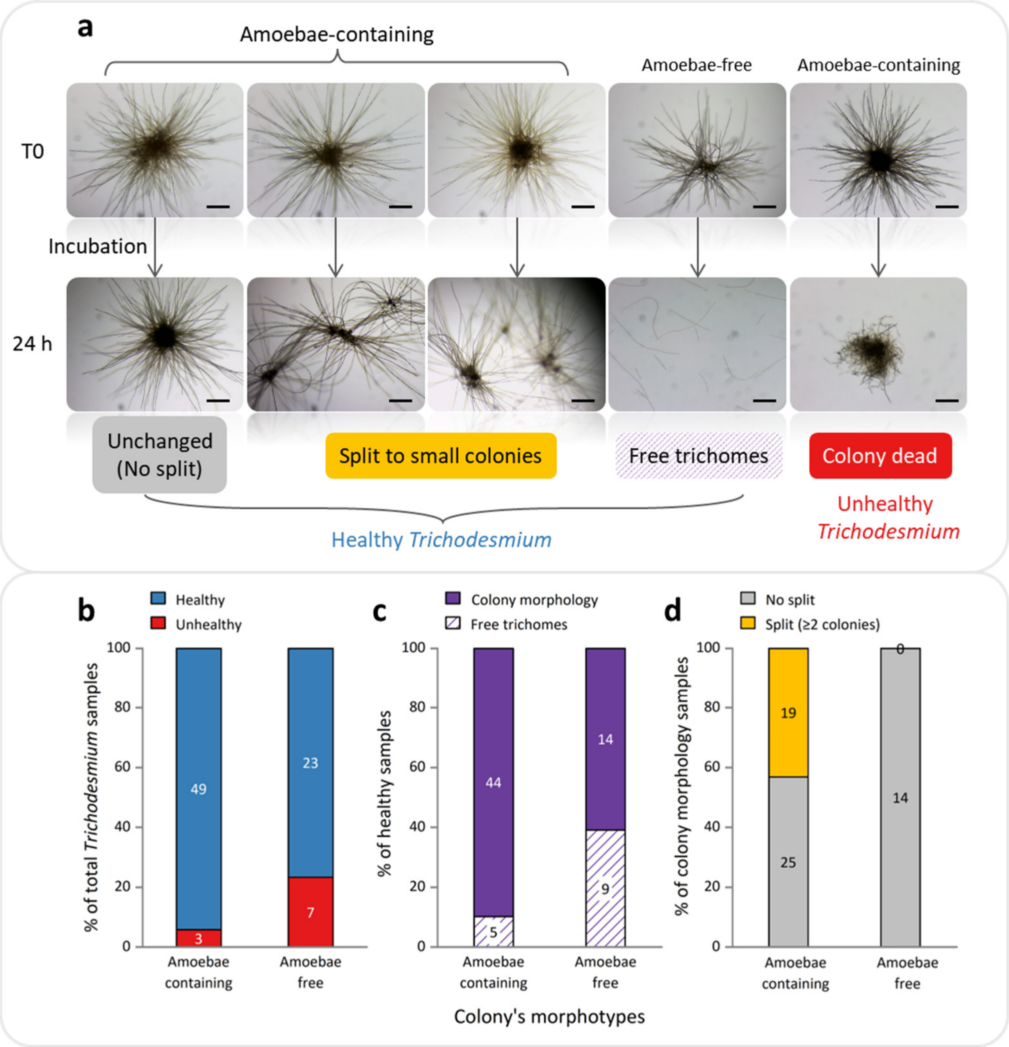

Figure S2. The effect of amoeba *Trichosphaerium micrum* on the health and morphology of Red Sea puff *Trichodesmium* colonies. Individual amoebae-containing and amoebae-free *Trichodesmium* colonies were incubated for 24 h in the laboratory. **(a).** Colonies were categorized based on their health condition and morphological changes. Individual colonies were observed and imaged before and after incubation using the stereoscope. Scale bars are 200 µm. **(b, c, d).** Fraction of different categories in two morphotypes of colonies. **(b).** Fraction of healthy and unhealthy samples in 24-hour incubated colonies. "Healthy" *Trichodesmium* samples were characterized by intact and dark-brown trichomes, while "Unhealthy" *Trichodesmium* samples included light green and broken trichomes. **(c).** Fraction of colony and free trichomes morphologies in the “healthy *Trichodesmium*” category from (b). The category “colony morphology” includes intact colonies and those split into more than one colony. **(d).** Fraction of “no split” and “split” in the category of “colony morphology” in (c).

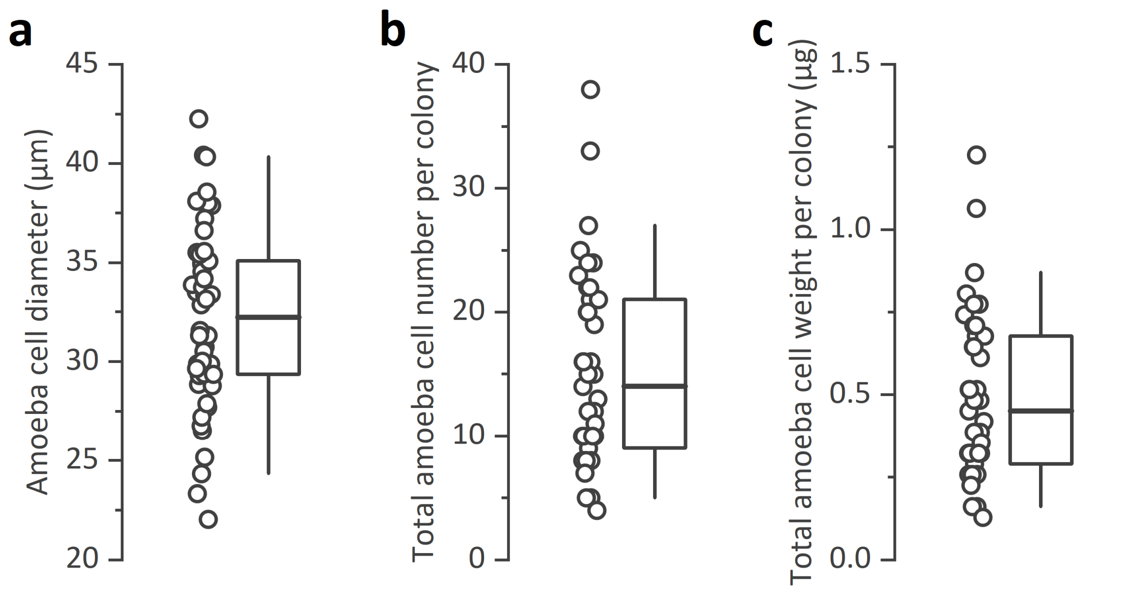

Figure S3. Boxplots depicting amoeba *Trichosphaerium micrum* characteristics within puff *Trichodesmium* colonies in the Red Sea. (a). Cell diameters of amoebae (n = 50) within fresh colonies. **(b, c).** The range of total cell number (b) and weight (c) of amoebae within individual colonies (n = 41). Total amoeba cell weight per colony was calculated by multiplying the corresponding total amoebae number by the average amoeba cell weight. The later was estimated based on its average cell density (1,848 ± 339 kg/m^3, n=5) and cell volume converted by cell diameter. Boxplots show the median, upper and lower quartiles and 1.5× interquartile range (IQR).

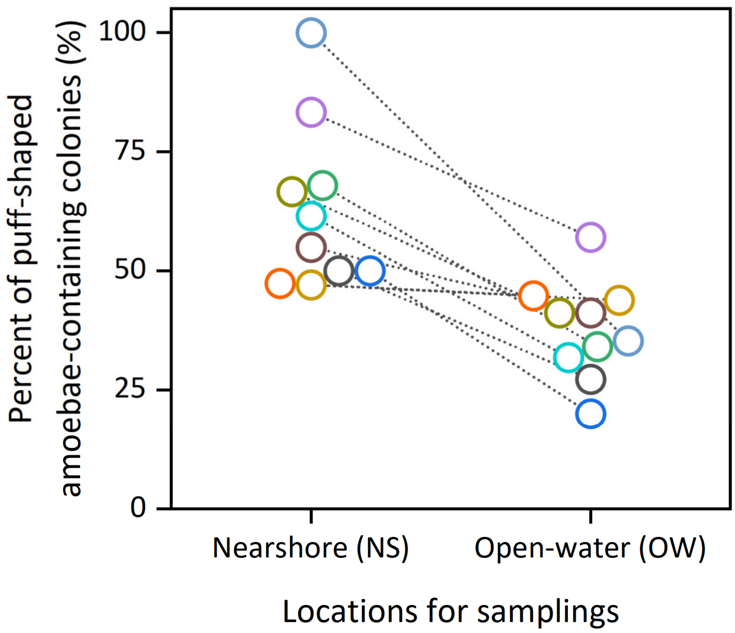

Figure S4. Spatial variations in the percentage of amoebae-containing colonies in the Red Sea. Colonies were collected from nearshore (NS) and open-water (OW) zones on the same sampling days during the spring of 2021. Open circles with the same color represent the same sampling day.

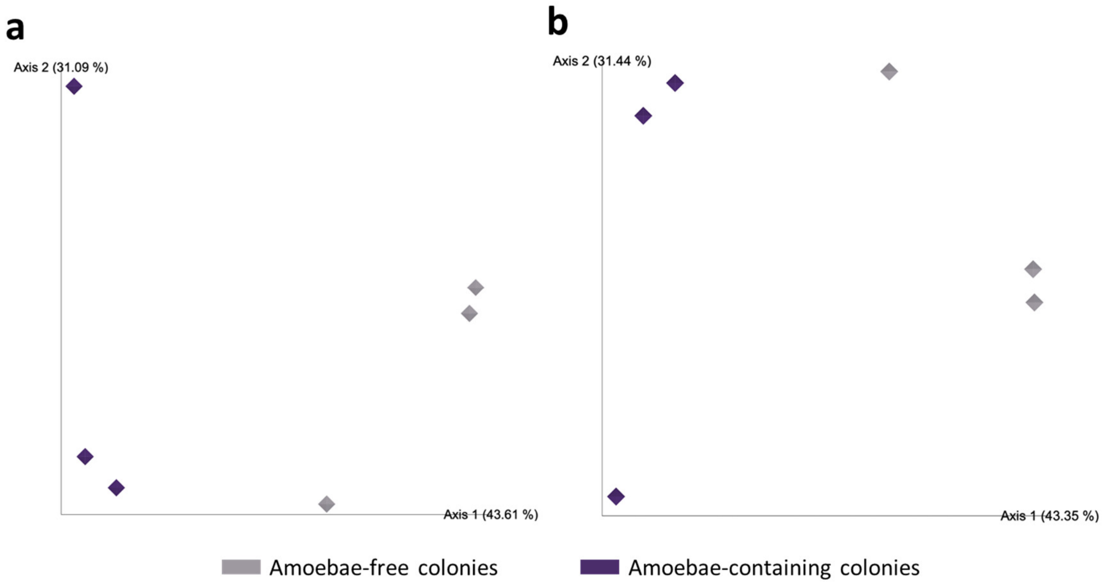

Figure S5. Principal components analysis (PCA) plots of Red Sea puff *Trichodesmium* colony 16S rRNA gene sequencing dissimilarity (beta-diversity, unweighted UniFrac distances). (a). all ASVs, with an average of 15,675 ASVs per sample. **(b).** ASVs excluding *Trichodesmium* ASVs, with an average of 8,316 ASVs per sample. Each point represents an individual sample, and greater distance indicates higher compositional dissimilarity. While samples are differentiated along Axis 1, there was no significant difference between amoebae-containing and amoebae-free colonies for a comparison of all ASVs (a, p=0.09) and non-*Trichodesmium* ASVs (b, p=0.10) using PERMANOVA with 999 permutations for unweighted UniFrac distance metrics.

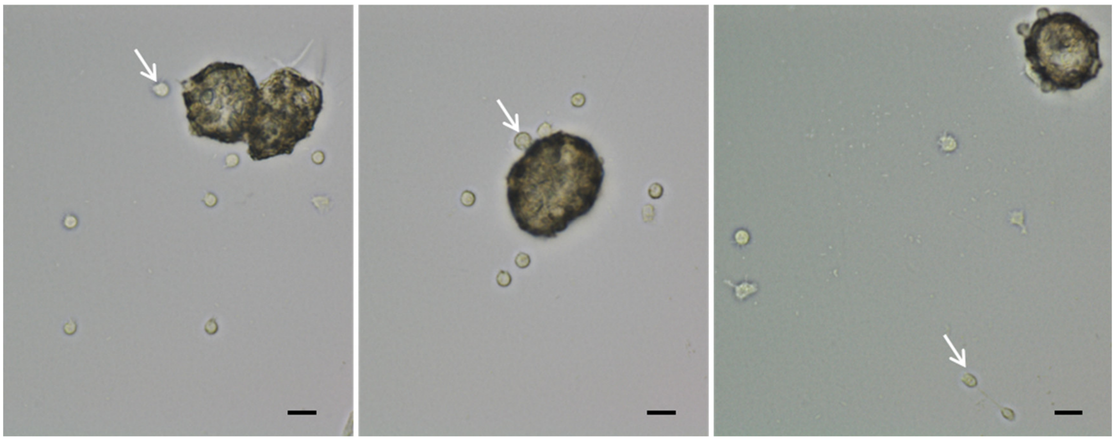

Figure S6. Schizogony of amoeba *Trichosphaerium micrum* cultured in hypersaline conditions. Small and spicule-free sporonts (arrows) released from spicule-coated *Trichosphaerium micrum* (testate form) [8]. Scale bars are 10 µm.

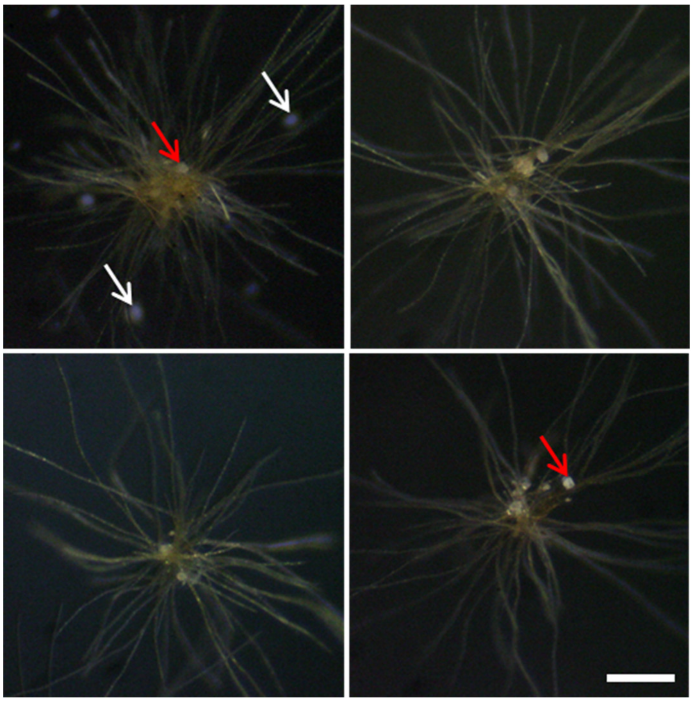

Figure S7. Association of active amoeba *Trichosphaerium micrum* with Red Sea puff *Trichodesmium* colonies in co-cultures on a shaker. Individual natural amoebae-free colonies were incubated with active amoebae in wells of 48-plates containing 1 mL FSW for 24 hours. Amoebae were observed attached to the colony's cores (red arrows) or the well bottom (white arrows). Scale bars are 200 µm.

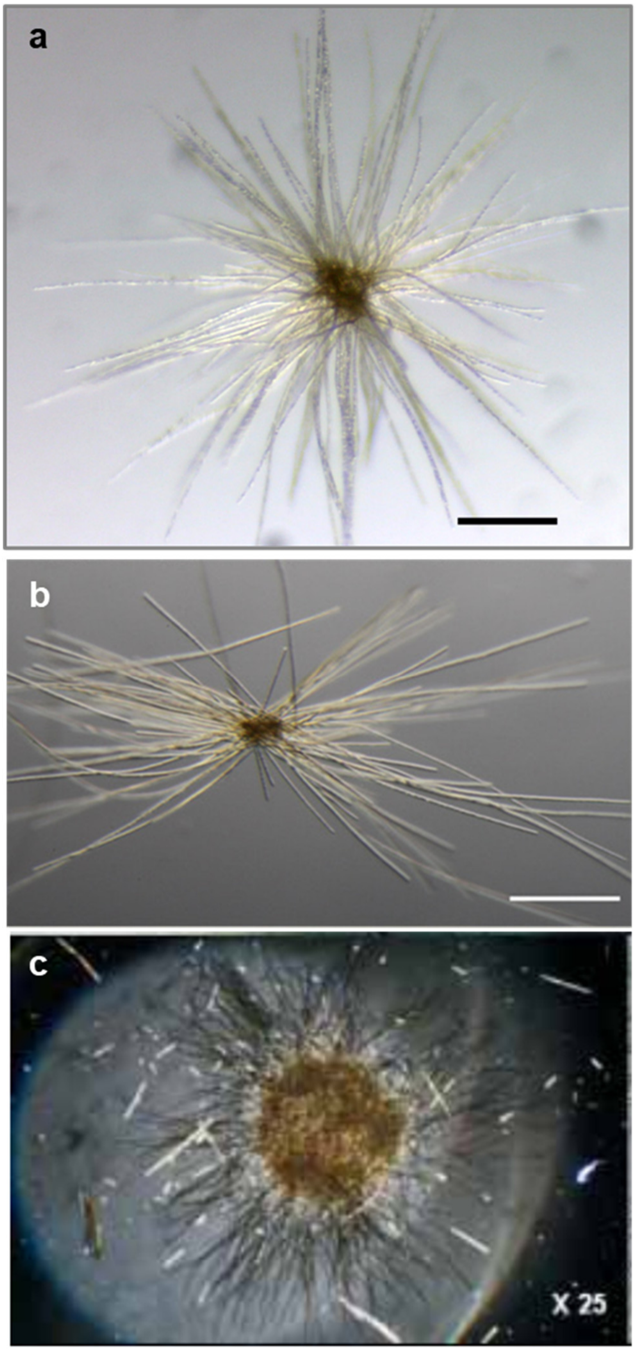

Figure S8. Natural puff *Trichodesmium* colonies with a dense core. (a). Amoebae-containing colony collected from the Red Sea. The colony core harbors amoebae (*Trichosphaerium micrum*). Scale bar = 200 µm. **(b).** Colony collected from the Great barrier reef. Image from [9], with permission, photographed by Julian Uribe-Palomino (CSIRO). Scale bar = 100 µm. **(c).** Colony collected from the Northeast New Caledonia coast. Image from [10], with permission.

4. References

1. Angell RW. Structure of *Trichosphaerium micrum* sp. n. J Protozool. 1975;22**:** 18-22.
2. Money NP, Fischer MW. What Is the Weight of a Single Amoeba and Why Does It Matter? The American Biology Teacher. 2021;83**:** 571-574.
3. Tandeau De Marsac N, Houmard J (eds). Complementary chromatic adaptation: Physiological conditions and action spectra. vol. 167, 1988.
4. Sheehan R, Banner FT. *Trichosphaerium*—An extraordinary testate Rhizopod from coastal waters. Estuarine and Coastal Marine Science. 1973;1**:** 245-260.
5. Angell RW. Observations on *Trichosphaerium platyxyrum* sp. n. J Protozool. 1976;23**:** 357-364.
6. Wang S, Zhang F, Koedooder C, Qafoku O, Basu S, Krisch S et al. Costs of dust collection by *Trichodesmium*: Effect on buoyancy and toxic metal release. Authorea Preprints. 2023.
7. White AE, Spitz YH, Karl DM, Letelier RM. Flexible elemental stoichiometry in Trichodesmium spp. and its ecological implications. Limnol Oceanogr. 2006;51**:** 1777-1790.
8. Schaudinn FR (eds). Untersuchungen Über den Generationswechsel von "*Trichosphaerium Sieboldi*" Schn. vol. Berlin Suppl. Abh. Konigl. Preuss. Akad. Wiss., 1899.
9. Davies C, Eriksen R, Richardson A (2020). Spatial and Seasonal trends in *Trichodesmium*. In: Richardson AJ, Eriksen R, Moltmann T, Hodgson-Johnston I, Wallis JR (eds). *State and Trends of Australia's Ocean (STAR)*. p 164.
10. Dupouy C, Dirgerg G, Tenório M, Neveux J, Le Bouteiller A. Surveillance des *Trichodesmium* autour de la Nouvelle-Calédonie, du Vanuatu, de Fidji et de Tonga 1998–2004. Archives Sciences de la Mer. 2004;7**:** 51.
